# The Action of Angiocrine Molecules Sourced from Mechanotransduction-Related Endothelial Cell Partially Explain the Successful of Titanium in Osseointegration

**DOI:** 10.3390/jfb14080415

**Published:** 2023-08-08

**Authors:** Patricia Fretes Wood, Célio Junior da Costa Fernandes, Gerson Santos de Almeida, Luisa Camilo Suter, João Paulo Ruiz Lucio de Lima Parra, Fábio José Bezerra, Willian Fernando Zambuzzi

**Affiliations:** Department of Chemical and Biological Sciences, Institute of Biosciences, UNESP—Universidade Estadual Paulista, Botucatu 18618-970, Brazil

**Keywords:** osteoblasts, endothelial cells, titanium, angiogenesis, mechanotransduction, osteogenesis, osseointegration

## Abstract

Since Branemark’s findings, titanium-based alloys have been widely used in implantology. However, their success in dental implants is not known when considering the heterogenicity of housing cells surrounding the peri-implant microenvironment. Additionally, they are expected to recapitulate the physiological coupling between endothelial cells and osteoblasts during appositional bone growth during osseointegration. To investigate whether this crosstalk was happening in this context, we considered the mechanotransduction-related endothelial cell signaling underlying laminar shear stress (up to 3 days), and this angiocrine factor-enriched medium was harvested further to use exposing pre-osteoblasts (pOb) for up to 7 days in vitro. Two titanium surfaces were considered, as follows: double acid etching treatment (w_DAE) and machined surfaces (wo_DAE). These surfaces were used to conditionate the cell culture medium as recommended by ISO10993-5:2016, and this titanium-enriched medium was later used to expose ECs. First, our data showed that there is a difference between the surfaces in releasing Ti molecules to the medium, providing very dynamic surfaces, where the w_DAE was around 25% higher (4 ng/mL) in comparison to the wo_DAE (3 ng/mL). Importantly, the ECs took up some of this titanium content for up to 3 days in culture. However, when this conditioned medium was used to expose pOb for up to 7 days, considering the angiocrine factors released from ECs, the concentration of Ti was lesser than previously reported, reaching around 1 ng/mL and 2 ng/mL, respectively. Thereafter, pOb exposed to this angiocrine factor-enriched medium presented a significant difference when considering the mechanosignaling subjected to the ECs. Shear-stressed ECs showed adequate crosstalk with osteoblasts, stimulating the higher expression of the Runx2 gene and driving higher expressions of Alkaline phosphatase (ALP), bone sialoprotein (BSP), and osteocalcin. Mechanotransduction-related endothelial cell signaling as a source of angiocrine molecules also stimulated the higher expression of the Col3A1 gene in osteoblasts, which suggests it is a relevant protagonist during trabecular bone growth. In fact, we investigated ECM remodeling by first evaluating the expression of genes related to it, and our data showed a higher expression of matrix metalloproteinase (MMP) 2 and MMP9 in response to mechanosignaling-based angiocrine molecules, independent of considering w_DAE or the wo_DAE, and this profile reflected on the MMP2 and MMP9 activities evaluated via gelatin-based zymography. Complimentarily, the ECM remodeling seemed to be a very regulated mechanism in mature osteoblasts during the mineralization process once both TIMP metallopeptidase inhibitor 1 and 2 (TIMP1 and TIMP2, respectively) genes were significantly higher in response to mechanotransduction-related endothelial cell signaling as a source of angiocrine molecules. Altogether, our data show the relevance of mechanosignaling in favoring ECs’ release of bioactive factors peri-implant, which is responsible for creating an osteogenic microenvironment able to drive osteoblast differentiation and modulate ECM remodeling. Taking this into account, it seems that mechanotransduction-based angiocrine molecules explain the successful use of titanium during osseointegration.

## 1. Introduction

In recent years, the bioactivity of dental implants has been researched with material development, and a number of studies have uncovered mechanical and biological properties predicted by novel materials in bone [1,2]. This shows the relevance of the biological properties of implants’ surfaces in mimicking natural tissues such as bone and, to this end, several bioactive surfaces have been proposed by creating a nanostructured coating using mimetically molecules, such as hydroxyapatite, or by modifying the topography of implants by subjecting them to acid treatment to ameliorate the roughness of the surface [1,3,4,5,6]. Both strategies are very welcome during implants’ interactions with host tissues, presenting good outcomes in dental rehabilitation when considering comorbidities [7,8]. Altogether, these ideas garner important value considering life expectancy worldwide and the increased levels of total and partial edentulism. Among implantable devices, titanium and its derivate alloys are mostly applied in both medical and dental surgeries to rehabilitate patients, given its ability to better associate with bone cells and molecules during the osseointegration process [5,9].

Although only titanium is currently used, very little is known about the relationships between bone cells, endothelial cells (ECs), and matrices during osteogenesis in the peri-implant microenvironment. In this scenario, it is expected that the healing process requires the activity of bone cells in crosstalking with ECs driving the morphogenetic changes in housing cells during osteogenesis. Thus, the connection between host bone tissue and the quality of the implant’s surface is crucial to mimic an ideal environment for healing [9,10,11]. However, osteoprogenitor cells are protagonists of this osseointegration mechanism, and very little value is credited to ECs and angiogenesis. Thus, the effect of housing ECs along the peri-implant microenvironment needs to be better evaluated in order to comprehend the harmony between cells and molecules. Angiogenesis deserves to be considered not only as a conduit for delivering cells and molecules, but also for playing a role in active paracrine signaling tissue during wound healing by recapitulating the earlier proposed interactions between them during bone growth [12]. We considered that bioinspiration in embryogenesis stages must be better applied during biomaterial development, looking for more biomimetic materials where the surface of biomaterials can initiate important biological changes [13,14,15,16]. In most cases, the cellular phenotype and quality of osseointegration are time-dependent, and it is consequently necessary to monitor the cellular response through biological players that control the intracellular pathways in response to the biomaterial surface [16,17,18,19,20]. In this scenario, the capacity of the biomaterial in adsorbing proteins on their surface to favor later cell adhesion is a desirable property for novel materials [21,22].

It is known that the cell adhesion process can occur in substrates and components of the extracellular matrix (ECM) upon extracellular integrins domains, which modifies its intracellular portion and creates points of interaction with intracellular proteins, such as Focal Adhesion Kinases (FAK) and Src [22,23,24,25,26,27,28]. This supramolecular platform promotes an adaptation in the cytoskeleton structures and establishes important parameters regarding the survival of cells, such as adhesion, proliferation, migration, and differentiation [29,30]. Considering focal adhesion kinase (FAK) and Src, the phosphorylation cascade is modulated by the capacity of those cells to guide the ECM components’ breakdown via specific matrix metalloproteinases and cathepsins activities, culminating immediately in the rearrangement of the cytoskeleton [9,11,31,32]. This mechanism, related to ECM remodeling, is expected during the implanted implants’ shelf-life, so the surface of titanium continuously releases signals able to activate cells to promote osteogenesis [32,33,34]. When the desirable osseointegration occurs, biocompatibility and osteo-inductivity processes will be reflected in close interactions between the cytoskeleton and biomaterial surface [35] during the bone remodeling periimplant. In this area, we have previously shown that titanium and its alloys are very dynamic materials and provide an important communication with surrounding tissues. Importantly, this mechanism, related to the dynamism of titanium, involves the modulation of enzymes able to control the phosphorylation balance intracellularly, such as the lower phosphorylation of PTP1B favoring the activity of FAK during osteoblast adhesion on dental titanium implants surfaces [36]. However, all of those findings open new perspectives to better mimic the periimplant microenvironment, considering the involvement of ECs on this context, and we have addressed here the hypothesis that ECs interfere in the activity of osteoblasts during the response to titanium in preliminary aspects of osseointegration.

Here, we investigated the influence of a titanium-enriched medium in the metabolism of ECs, and thereafter its effect on osteoblasts was also considered. This is expected to build on proposed novel in vitro strategies as alternative methodologies able to minimize the use of experimentation-related animals, where angiocrine-released factors from EC responding to titanium can be used to expose pre-osteoblasts (pOb). Considering a very similar strategy, we have better addressed the effect of Cobalt-Chromium in this regard, and it was valid [37]. Furthermore, the effect of angiocrine factors in paracrine-signaling-dependent osteogenesis required during the osseointegration of dental implants was considered. In this study, we have inaugurated the term “angiocrine molecules”, referring to molecules released by endothelial cells responding to titanium-conditioned medium and considering the shear stress mimicking the blood flow. Technically, we have also considered culturing the ECs responding to laminar shear stress, looking to mimic the tensional forces of blood flow with respect to physiological mechanotransduction-related endothelial cell signaling.

## 2. Materials and Methods

### 2.1. Materials and Titanium Alloys

Titanium alloy discs were obtained from S.I.N. (São Paulo, SP, Brazil). Cell culture staffs were obtained from TPP Techno Plastic Products AG (Trasadingen, Switzerland), and the cell culture media for cell experiments were purchased from Nutricell Co. (Campinas, São Paulo, Brazil). For qPCR technology, primers were obtained from Exxtend Oligo Solutions (Paulinia, São Paulo, Brazil) and GoTaq^®^ qPCR Master Mix (A6002) was obtained from PROMEGA (Madison, WI, USA). All analytical-grade chemicals were purchased from Sigma-Aldrich (St. Louis, MO, USA).

### 2.2. Cell Culture

**Figure 1:**
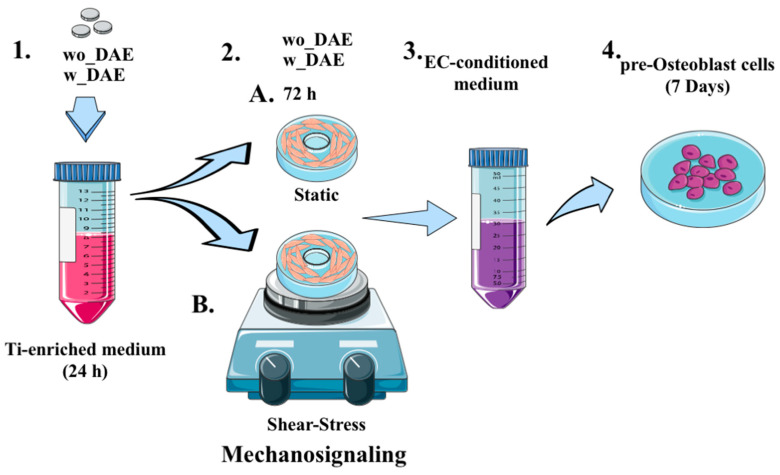
Schematization of the experimental flow and timeline. In order to better understand the relevance of endothelial cells (ECs) on osteoblasts responding to titanium-enriched medium, a new model was proposed by incubating the body-of-proof (discs) of titanium subjected to two different surfaces: presenting DAE surface (w_DAE) and machined surface (wo_DAE). The discs were maintained in cell culture medium in conical tubes (**1**) for up to 24 h in cell culture incubator. Thereafter, the Ti-enriched medium was used to expose ECs for up to 3 days, with respect to 2 experimental conditions: static (A), where ECs were maintained in conventional cell culture conditions, and dynamic (B), where ECs were maintained considering shear-stress. In both conditions, the EC cultures were exposed to Ti-enriched medium (**2**). The EC-conditioned medium was harvested after 3 days (**3**), centrifuged, and later used to expose pre-osteoblasts (pOb) for up to 7 days (**4**). Both the cell culture medium and pObs were harvested separately to later evaluate MMP’s activity and transcriptional profile of specific genes, respectively. Servier Medical Art models, licensed under a Creative Commons Attribution 3 License. Not ported; https://smart.servier.com (accessed on 1 August 2023).

HUVECs (Human Umbilical Vein Endothelial Cells; also named ECs) and pre-osteoblasts (MC3T3-E1, subclone 4; pOb) were purchased from American Type Culture Collection-ATCC (Rockville, MD, USA; passage < 20) and certified as mycoplasma-free cells. Routinely, the lineages were incubated at 37 °C and 95% humidity in an environment containing 5% of CO_2_. The specific cells were cultured respecting the manufacturer’s recommendations and the cell culture medium was supplemented with 1% of antibiotics (100 U/mL penicillin, 100 mg/mL streptomycin) and 10% Fetal Bovine Serum (FBS) (Nutricell, Campinas, SP, Brazil). The experimental workflow, considering the response of osteoblasts to angiocrine molecules and titanium, was as follows: (1) control (Ctrl)—pOb cultures maintained in a traditional supplemented α-MEM medium and O.M. (Osteogenic Medium), where osteoblasts were maintained under known osteogenic medium stimulant (osteoblast differentiation positive control); (2) osteoblasts exposed to conditioned medium by ECs (both shear-stressed or not), here also considered a reference group for analyzing the effect of titanium; and (3) pObs exposed to conditioned medium from non-shear stressed ECs responding to titanium. Figure 1 (items 1–4, top of the panel) summarizes the experimental workflow used in this study. Different titanium-modified surfaces were investigated; w_DAE: double acid-ecthed (w_DAE) or wo_DAE: without DAE.

### 2.3. Preparation of Angiocrine Molecules-Obtaining Medium

Initially, titanium discs were incubated within Falcon^®^ Conical Centrifuge Tubes (Glendale, AZ, USA) containing FBS-free cell culture medium for 24 h, as recommended by ISO 10993:2016, and the samples were properly centrifuged to avoid any debris and later used to evaluate the behavior of cells (Figure 1). It was here expected that the surface named DAE should release titanium molecules. Furthermore, ECs were exposed to that titanium-enriched medium for 3 days, when the EC-conditioned medium containing angiocrine molecules samples was duly collected to allow the analysis of the titanium content. It is important to consider here that ECs were subjected to shear stress as a testing group, as well as considering non-stressed cells as a comparable and reference group, avoiding the mechanical factors described to mimic hemodynamic forces of physiological blood flow (Figure 1). Both media conditioned by ECs were considered in this study to contain angiocrine molecules, soluble molecules released by ECs from the metabolism in response to soluble titanium. Finally, the angiocrine molecules containing the medium were later used to expose osteoblasts for 7 days, and then the cell samples were harvested to allow for the molecular analysis.

### 2.4. The Laminar Shear-Stress Model Was Applied to Mimic Mechanotransduction-Related Endothelial Cell Signaling Related to Blood Flow

Modified cell culture dishes were previously prepared with medical silicone, bonding smaller 60 mm diameter cell culture dishes in the center of a 100 mm diameter. These modified cell culture dishes were later sterilized with a UV light for up to 30 min prior to use along the experimental workflow and timeline. It is important to mention that any cytotoxicity was detected by cells’ exposure to medical silicone. Semi-confluent (around 85%) monolayers of ECs were cultured in the peripheral ring of the modified dishes’ area to simulate blood flow, mimicking the laminar tensional forces obtained from an orbital shear-stress modulus, as previously reported [38,39,40,41]. All experiments were conducted with an orbital shaker (Scilogex, Rocky Hill, CT, USA) in a CO_2_ incubator at 37 °C and 5% CO_2_ atmosphere. The calculation of shear stress was described earlier (Pinto et al., 2019).

### 2.5. Titanium Content Was Quantified with GFAAS

For titanium quantification (Ti), 20 μL of culture medium was further used for the analysis, as depicted in Figure 1. The conditioned culture medium was used to expose ECs, and aliquots were collected to evaluate. Next, the Ti-enriched medium was later used to expose ECs for up to 3 days, and thereafter the medium carried the angiocrine molecules to osteoblasts for up to 7 days. At each stage, aliquots were saved to measure the titanium content in order to infer some conclusions about the potential uptake by the cells. All samples were measured in the GFAAS technology using a SHIMADZU model AA-6800 atomic absorption spectrometer. Such a spectrometer has a background absorption module attached in a self-reverse system (SR) with a pyrolytic graphite tube integrated platform and an ASC-6100 automatic sampler. A Shimadzu hollow cathode lamp of Ti operated at a minimum current of 12 mA and a maximum current of 600 mA (currently used in background correction—BG). Argon was used as the inert gas, and a constant flow of 1 L min^−1^ was maintained during the entire heating program, except for the atomization stage, during which the gas flow was stopped. The absorbance measurements (based on the peak area) were carried out in triplicate. The graphite tube’s heating program, optimized for titanium determination, was based on the procedure described by Silva et al. [42] with some modifications, and it is described as follows: drying temperature—120/250 °C; pyrolysis temperature—900 °C; atomization temperature—900 °C; and cleanup temperature—2600 °C.

### 2.6. mRNA Isolation and qPCR Analysis

Osteoblasts cultures responding to different conditions detailed here earlier were properly collected using the trizol method, exactly as recommended by the manufacturer (Life Sciences, Thermo Fisher Scientific Inc., Waltham, MA, USA), where the total RNA was carried out by adding DNase I (Invitrogen, Carlsbad, CA, USA) and the measuring was performed in a Biotek microplate reader (Synergy multi-mode reader, Biotek, TX, USA). cDNA was synthetized by using a High-Capacity cDNA Reverse Transcription Kit (Applied Biosystems, Foster City, CA, USA). qPCR was performed by evaluating the expression of the genes which are listed in Table 1, in a total of 10 μL, containing PowerUpTM SYBRTM Green Master Mix 2x (5 μL; Applied Biosystems, Foster City, CA, USA), 0.4 μM of each primer, 50 ng of cDNA, and nuclease-free water. The graphs show the relative amounts of the transcripts using glyceraldehyde 3-phosphate dehydrogenase (GAPDH) as a housekeeping gene, using the cycle threshold (Ct) method.

### 2.7. Scanning Electron Microscopy (SEM) and Energy Dispersive X-ray (EDX)

Osteoblasts and ECs were cultured directly on the titanium-based surfaces, and the morphological changes were monitored for up to 3 days. SEM images were acquired and EDX analysis was performed by subjecting the samples to scanning electron microscopy using FEI (Quanta 200; Hillsboro, Oregon, EUA), attached to the secondary electrons detector. The individual elements’ composition of materials (carbon and titanium) was analyzed with an Energy Dispersive X-ray (EDX) module using a proper EDX detector from Oxford INCA 250p20. All collected data had a semi-quantitative profile determined by evaluating 3 different areas of the same sample, which was correlated between the 3 measurement averages at 20 kV energy operation. Field-of-view was defined by 500 times’ magnification.

### 2.8. Gelatin Proteolysis-Based Zymography

During the experimental model, aliquots of the medium conditioned by ECs or osteoblasts were harvested to further estimate the activity of matrix metalloproteinases (MMP) using a widely explored gelatin proteolysis-based zymography. In order to avoid the debris, the samples were properly centrifuged by applying 14,000 rpm for up to 15 min and the protein amount was assayed using the Lowry protocol [43]. The previously quantified protein was later determined to guide the load of the pool of the proteins resolved using a 12% polyacrylamide gel containing 4% gelatin, and the renaturation of proteins was performed by using Triton X-100 aqueous solution (2% *w*/*v*) for up to 18 h in proteolysis buffer (Tris—CaCl_2_) at 37 °C and then staining with Coomassie Blue R- 250 dye solution 0.05% for up to 3 h. The stained gels were washed in 30% methanol (*v*/*v*) and 10% glacial acetic acid solution (*v*/*v*) containing solution. The bands were considered to measure the gelatinolytic activity of metalloproteinases 2 (MMP2, ~62 kDa) and 9 (MMP9, ~84 kDa), and then they were subjected to the densitometric analysis using the software ImageJ (Bethesda, MD, USA) [44].

### 2.9. Statistical Analysis

The replicates of each experiment were represented as mean ± standard error deviation (SE) (n = 3). Significant differences were when *p* < 0.05. The statistical data were collected using analysis of variance (two-way ANOVA) with multiple comparisons of all pairs of groups, applying Bonferroni’s correction posttest, or nonparametric analysis. Further, the significance level was considered when alpha = 0.05. All data were obtained using GraphPad Prism 7 program (GraphPad Software, La Jolla, CA, USA).

## 3. Results

### 3.1. Dynamic Interaction of Titanium Surface with the Microenvironment

**Figure 2:**
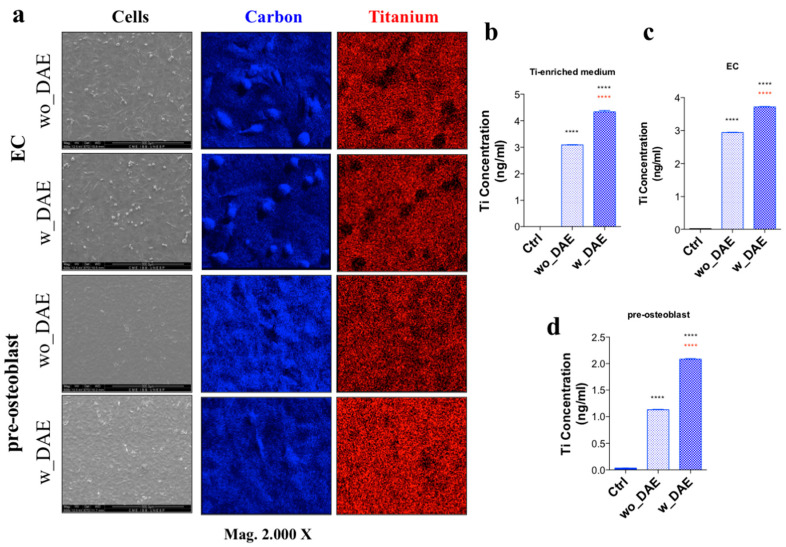
Titanium surfaces dynamically interact with the microenvironment. Cells were cultured directly on both titanium-based surfaces, wo_DAE and w_DAE, for up to 3 days and technically prepared by SEM analysis (**a**). Additionally, carbon and titanium elements’ distribution on the surfaces were also investigated using EDX analysis. Magnification: 2.000×. Also, the released titanium was monitored in the conditioned medium, and was around 3 ng/mL and 4 ng/mL from wo_DAE and w_DAE, respectively (**b**). This Ti-enriched medium was afterwards used to expose ECs for up to 3 days, when a new aliquot was measured considering the Ti amount; it was not significant (**c**). However, when these angiocrine molecules containing medium were later used to expose osteoblasts for up 7 days, there was an uptake by those cells of around 2 ng/mL in each group investigated, decreasing to around 1 ng/mL in wo_DAE and around 2 ng/mL in w_DAE (**d**). **** *p* < 0.0001 was considered a significant difference (“black” when the test groups (both wo_DAE and w_DAE) were compared with the control (Ctrl) and “red” when the test groups were compared each other).

We investigated the morphology of ECs and osteoblasts directly interacting with both wo_DAE and w_DAE surfaces for up to 3 days. SEM images showed expected morphological changes in both cells (Figure 2a). Additionally, these samples were subjected to EDX analysis, through which it was possible to map the distribution of carbon and titanium’s spread on the evaluated surfaces (Figure 2a). Thereafter, to determine whether the release of titanium from the titanium-based alloys was significant, we used GFAAS technology. Briefly, the discs were maintained in a conical tube in FBS-free cell culture medium for 24 h at 37 °C, when the titanium-enriched medium was harvested and subjected to analysis. Our data showed that w_DAE (~4 ng/mL) released a higher amount of titanium than wo_DAE (~3 ng/mL) (Figure 2b), and this difference might be in relation to the roughness promoted by DAE. Thereafter, this medium was further used to expose ECs, and Figure 2c brings clear evidence that there was no significant uptake of this released titanium by ECs for up to 3 days of exposition; the amount of soluble titanium found in the cell culture medium was very similar to that shown earlier the exposition, with ~4 ng/mL for w_DAE, and ~3 ng/mL for wo_DAE. Conversely, the osteoblasts had an uptake of ~2 ng/mL for up to 7 days of exposition, independently of w_DAE or wo_DAE (Figure 2d).

### 3.2. In Conjunction, Angiocrine Soluble Factors and Titanium Promote Osteogenic Gene Markers’ Requirements

**Figure 3:**
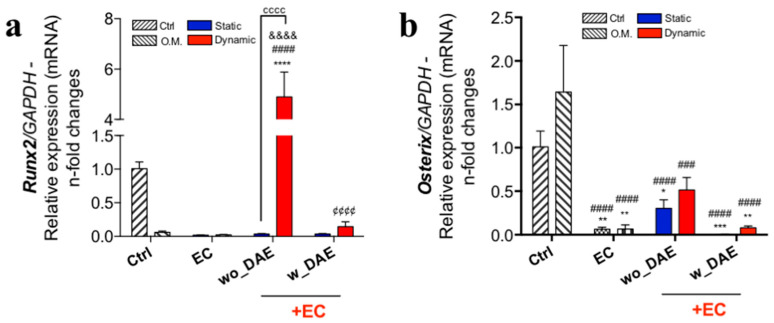
Osteogenic transcription factors’ Runx2 and Osterix genes are modulated by angiocrine molecules. We evaluated whether angiocrine molecules from ECs participate in the osteoblast response to titanium-enriched medium. Osteoblasts samples were harvested and later used to evaluate the activation of transcription factors’ Runx2 (**a**) and Osterix (**b**) genes. Both genes were higher in osteoblasts exposed to angiocrine factor sourced from mechanotransduction-related endothelial cell signaling, independently of wo_DAE or w_DAE. However, regarding Runx2, it seems that wo_DAE developing a better performance. The experimental workflow brings the analysis of different control cultures, as follows: Ctrl: cultures were maintained under conventional condition; O.M.: cultures were under osteogenic medium condition; EC: osteoblasts were exposed to angiocrine molecules obtained by EC (endothelial cell) under shear stress (dynamic) or non-subjected to SS (static). The qPCR running data of specific genes were later normalized by using GAPDH as a housekeeping gene. Statistics were considered significant when * *p* = 0.0223; ** *p* = 0.0014; *** *p* = 0.0007; ^###^ *p* = 0.0008; **** *p* < 0.0001; ^####^ *p* < 0.0001; ^&&&&^ *p* < 0.0001; ^¢¢¢¢^ *p* < 0.0001; and ^cccc^ *p* < 0.0001.

The peri-implant microenvironment requires a synchrony of events (involving molecular and cellular issues), able to drive osteogenesis and consequently accelerating the osseointegration of implants. Although titanium has been widely used in biomedical applications, the molecular and cellular crosstalk governing its biocompatibility in bone needs to be better addressed. Here, we have now investigated whether ECs impact the titanium effect on the osseintegration process by paracrine modulating the osteoblast’s phenotype. Firstly, in this sense, it was shown that the angiocrine soluble factors-containing medium was able to activate classical transcription factors of the early osteogenic phenotype mechanism. Importantly, the relevance of mechanotransduction-related endothelial cell signaling in releasing those angiocrine molecules able to reach significance regarding both Runx2 and Osterix genes is shown here. Runx2 is a master switch for inducing osteoblast differentiation. Additionally, this response was even more significant considering the mechanotransduction-related endothelial cell signaling response to wo_DAE (Figure 3a,b).

**Figure 4:**
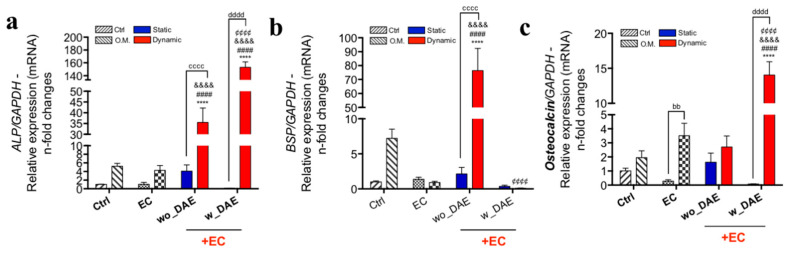
Differentiated osteoblast gene markers were higher in response to angiocrine factors obtained from EC. Again, the hypothesis of angiocrine factors contributing to the titanium response looking for the osteogenic phenotype of osteoblasts was investigated considering the expression of genes related to late stages of osteoblast differentiation. Thus, alkaline phosphatase (ALP) (**a**), bone sialoprotein (BSP) (**b**) and osteocalcin (**c**) genes were properly investigated in this way. Although presenting very similar responses to mechanotransduction-related endothelial cell signaling, BSP also seemed to consider the effect of wo_DAE. The experimental workflow enabled the analysis of different control cultures, as follows: Ctrl: cultures were maintained under conventional condition; O.M.: cultures were under osteogenic medium condition; EC: osteoblasts were exposed to angiocrine factors obtained by EC (endothelial cell) under shear stress (dynamic) or non-subjected to SS (static). The qPCR running data of specific genes were later normalized by using GAPDH as a housekeeping gene. Statistics were considered significant, when: **** *p* < 0.0001; ^####^ *p* < 0.0001; ^&&&&^ *p* < 0.0001; ^¢¢¢¢^ *p* < 0.0001; ^bb^ *p* = 0.0069; ^cccc^ *p* < 0.0001; and ^dddd^ *p* < 0.0001.

Thereafter, until considering the osteogenic phenotype, genes related to final events of osteoblasts’ differentiation were also investigated in response to the titanium-conditioned medium in association with angiocrine molecules, mature osteoblasts expressing alkaline phosphatase (ALP), bone sialoprotein (BSP), and osteocalcin (OCN). Regarding ALP gene activation, our data clearly showed that there is an influence of ECs on osteoblasts responding to titanium-enriched medium. Importantly, both surfaces promoted higher ALP expression when titanium was associated with angiocrine molecules released by shear-stressed ECs, strongly suggesting the mechanotransduction-related endothelial cell signaling in supporting ALP gene expression in osteoblasts. Here, there was a significant difference considering both evaluated surfaces; in response to wo_DAE, the ALP gene expression was ~35-fold changes higher, while w_DAE promoted, surprisingly, ~150-fold changes higher when compared to the control (Ctrl) (Figure 4a). Importantly, O.M. promoted a higher expression of the ALP gene, and it could be considered in the validation of this biological model. Additionally, the ALP expression profile shown here was very similar to that found considering osteocalcin gene expression (Figure 4c). Specific to osteocalcin, shear-stressed EC-obtained angiocrine molecules (without association with titanium) were sufficient to promote higher expressions of osteocalcin.

The bone sialoprotein (BSP) gene was also investigated in this context, and a very similar profile was found regarding Runx2 gene behavior. Mechanotransduction-related endothelial cell signaling seemed to contribute actively as a source of angiocrine molecules, which is necessary to preserve an osteogenic microenvironment, mainly considering the association with titanium-enriched medium by wo_DAE (Figure 4b).

### 3.3. Mechanotransduction-Related Endothelial Cell Signaling Drives Angiocrine Molecules Requiring Col3A1 and the Profile of ECM Remodeling in Differentiating Osteoblasts

**Figure 5:**
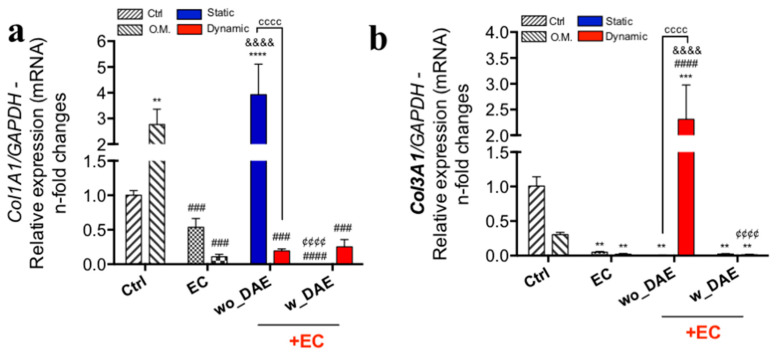
Col3A1 gene was higher in response to mechanotransduction-related endothelial cell signaling sourced angiocrine molecules. Considering the bone ECM synthesis, in response to angiocrine molecules, Col1A1 (**a**) and Col3A1 (**b**) genes were evaluated. Our data showed an enhanced expression of both genes in response to wo_DAE. The experimental workflow enables the analysis of different control cultures, as follows: Ctrl: cultures were maintained under conventional condition; O.M.: cultures were under osteogenic medium condition; EC: osteoblasts were exposed to angiocrine factors obtained by EC (endothelial cell) under shear stress (dynamic) or non-subjected to SS (static). The qPCR running data of specific genes were later normalized by using GAPDH as a housekeeping gene. Statistics were considered significant, when: ** *p* = 0.0086; *** *p* < 0.0005; **** *p* < 0.0001; ^###^ *p* = 0.0008; ^####^ *p* < 0.0001; ^&&&&^ *p* < 0.0001; ^¢¢¢¢^ *p* < 0.0001; and ^cccc^ *p* < 0.0001.

As osteoblasts are responsible for growing the new bone, and intermediating the synthesis of organic and inorganic portions of the bone extracellular matrix (ECM), we also investigated the expression of genes that are necessary and sufficient to induce the mineralization of this ECM. Here, we have complimentarily evaluated the capacity of angiocrine molecules in association with titanium in trigger intracellular signaling in osteoblasts able to drive the expression of Col1A1 (Figure 5a) and Col3A1 genes (Figure 5b). Considering the Col1A1 gene, it was higher in O.M., as expected, and was ~5-fold changes higher in the angiocrine factor released in response to the static condition of ECs responding to wo_DAE, suggesting that it is not dependent on mechanotransduction-related endothelial cell signaling. In this sense, the osteoblasts responding to EC also presented higher expressions of the Col1A1 gene. Conversely, the Col3A1 gene was dependent on mechanotransduction-related endothelial cell signaling, obtaining angiocrine factors in response to wo_DAE; it reached ~2.5-fold changes higher than the control (Crtl) (Figure 5b). 

**Figure 6:**
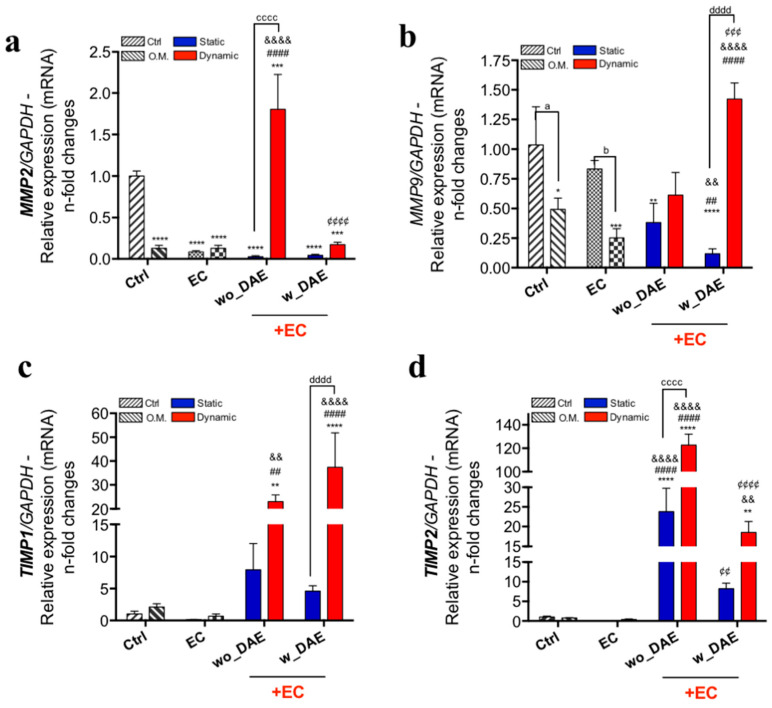
Bone ECM remodeling-related gene markers are finely modulated via mechanotransduction-related endothelial-cell-signaling sourced angiocrine soluble molecules. Here, some advancement was achieved considering the bone ECM remodeling via evaluating matrix metalloproteinases (MMP)2 (**a**) and MMP9 (**b**), and the tissue inhibitor properties of metalloproteinases 1 (TIMP-1) (**c**) and TIMP2 (**d**) genes were also investigated. The experimental workflow allows the analysis of different control cultures, as follows: Ctrl: cultures were maintained under conventional condition; O.M.: cultures were under osteogenic medium condition; EC: osteoblasts were exposed to angiocrine molecules obtained by EC (endothelial cell) under shear stress (dynamic) or non-subjected to SS (static). The qPCR running data of specific genes were later normalized by using GAPDH as a housekeeping gene. Statistics were considered significant when * *p* = 0.0223; ** *p* = 0.0086; *** *p* = 0.0005; **** *p* < 0.0001; ^##^ *p* = 0.0027; ^####^ *p* < 0.0001; ^&&^ *p* = 0.0035; ^&&&&^ *p* < 0.0001; ^¢¢^ *p* < 0.0011, ^¢¢¢¢^ *p* < 0.0001; ^a^ *p* = 0.0208; ^b^ *p* = 0.0117; ^cccc^ *p* < 0.0001; and ^dddd^ *p* < 0.0001.

**Figure 7:**
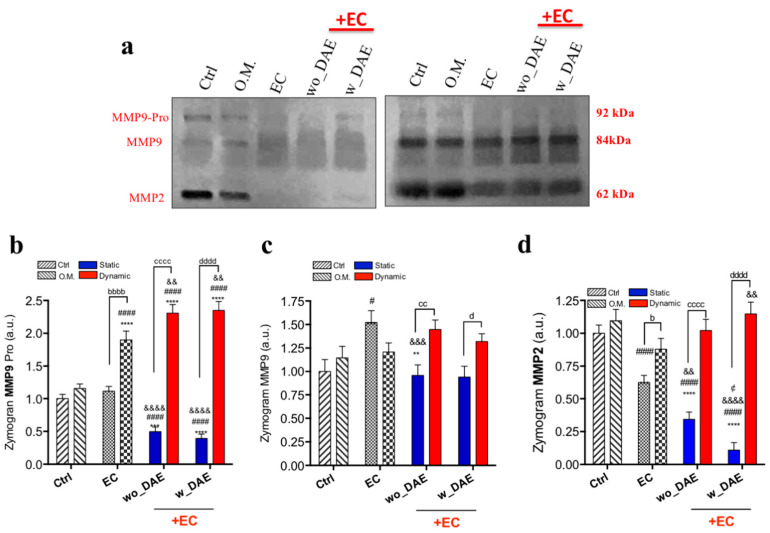
MMPs activities in Ob are modulated by angiocrine soluble molecules sourced from mechanotransduction-related endothelial cell signaling. Complimentarily, MMP2 (**d**) and MMP9’s (**b**,**c**) activities were investigated considering a gelatin proteolysis-based zymography, as it displayed a representative zymogram (**a**). The experimental workflow allows the analysis of different control cultures, as follows: Ctrl: cultures were maintained under conventional condition; O.M.: cultures were under osteogenic medium condition; EC: osteoblasts were exposed to angiocrine molecules obtained by EC (endothelial cell) under shear stress (dynamic) or non-subjected to SS (static). Statistics were considered significant, when: ** *p* = 0.0026; *** *p* = 0.0006; **** *p* < 0.0001; ^#^ *p* = 0.0406; ^####^ *p* < 0.0001; ^&&^ *p* = 0.0065; ^&&&^ *p* = 0.0003; ^&&&&^ *p* = 0.0001; ^¢^ *p* = 0.0324; ^b^ *p* = 0.0018; ^bbbb^ *p* < 0.0001; ^cc^ *p* = 0.0018; ^cccc^ *p* < 0.0001; ^d^ *p* = 0.0209; and ^dddd^ *p* < 0.0001.

Also, the balance of expression of genes related to ECM remodeling was evaluated by checking the expression of MMPs and TIMPs (Figure 6). The MMPs gene was significantly higher (~1.5-fold changes; Figure 6) in response to wo_DAE in conjunction with angiocrine molecules obtained in shear-stressed ECs, while conversely the MMP9 gene developed a different effect, here being higher in osteoblasts exposed to w_DAE in conjunction with shear-stressed ECs (Figure 6b). MMP2 gene activation seemed to reflect the profile of Col3A1 gene behavior. Additionally, both TIMP1 (Figure 6c) and TIMP2 (Figure 6d) genes were also higher in response to angiocrine factors from shear-stressed ECs independently when exposed to wo_DAE or w_DAE. Finally, MMPs’ activities were also evaluated using gelatin proteolysis-based zymography (Figure 7a), and both profiles of those MMPs directly reflected the gene expression of both MMP2 (Figure 7d) and MMP9 (Figure 7b,c), with the significant effect of ECs releasing angiocrine soluble molecules in synergy with the titanium-enriched medium.

**Figure 8:**
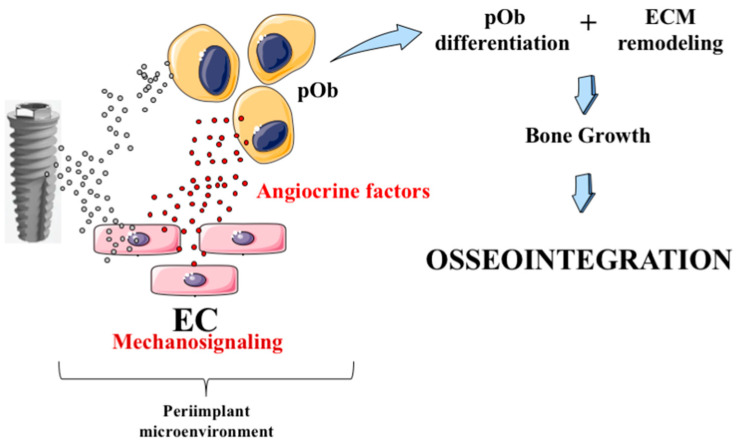
Schematization chart gathers the main findings of this study, explaining the biocompatibility of titanium-based alloys. The titanium released from dental implants affects the biology of endothelial cells, which release angiocrine molecules under mechanotransduction-related endothelial cell signaling. This study shows these angiocrine molecules promoting morphogenetic changes in osteoblasts, resulting in the maturation of their differentiation mechanisms with higher expressions of osteogenic gene markers and their capacity in remodulating bone ECM. Altogether, these findings might explain the performance of titanium in bone, where the angiocrine molecules sourced from mechanotransduction-related endothelial cell signaling are the protagonists in this scenario.

Finally, Figure 8 summarizes the main findings found in this study, where the protagonist of mechanotransduction-related endothelial cell signaling displays relevance in osteoblast differentiation throughout its different stages, also compromising bone ECM remodeling. Altogether, by gathering these data, we have a draw of the osseointegration biology promoted by titanium alloys.

## 4. Discussion

Titanium has so far been the gold-standard material within the biomaterial development field considering osseointegration. Although some progress has been achieved over the last years on this issue, there is still a lack of advancement in comprehending the crosstalk among housing cells and molecules during bone growth and tissue healing, which might bring light when explaining the biology of osseointegration. Over the last decade, we have proposed in vitro models to better understand the behavior of cells and molecules when interacting with biomaterials, since ceramic to metallic devices and a map of molecules have been gathered to predict the better performance of cells in desirable materials and their surfaces [1,45,46,47,48,49]. In this study, we addressed the gap in knowledge about the synchrony of cells and molecules during osseointegration by applying an in vitro biological model able to evaluate the potential communication between endothelial cells (ECs) and osteoblasts, mainly motivated by evaluating whether the pioneer findings by Kusumbe et al. (2014) were recapitulated during the bone healing surrounding dental implants [12]. They have sufficiently shown that a specific EC physiologically drives the osteogenesis by assessing NOTCH signaling [50]. In this way, our data show the relevance of laminar-shear-stress suffering ECs on guiding osteoblast differentiation responding to titanium released from dental implants. Furthermore, our data also suggest a protagonist for mechanotransduction-related endothelial cell signaling as a source of bioactive molecules (angiocrine factors) able to coordinate bone healing across the osseointegration mechanism.

Technically, in this direction, we firstly evaluated the concentration of titanium released from dental implants for up to 24 h, and our data suggested a very dynamic microenvironment actively interacting with the dental implants’ surfaces. This is the time to mention that a titanium-enriched medium was previously shown to coordinate the activity of bone and endothelial cells [18,20,33,51,52]. Previously, we have shown the ability of this Ti-released amount in modulating the intracellular activity of protein tyrosine phosphatase 1B (PTP1B) and its results on the higher phosphorylation of Focal Adhesion Kinases (FAK), and it reinforces its capacity in interfering in osteoblast adhesion. This molecular draw depicts a relevance to Reactive Oxygen Species (ROS) in oxidizing cysteines at the active site of PTPs and interference in the phosphorylation balance in cells [36,53]. Now, our data bring forward a new discussion in this regard; once a slight amount of titanium has been uptaken by ECs, it might contribute to releasing angiocrine factors. However, more results need to be found, mainly considering the effect of ROS on the machinery able to express and release those angiocrine molecules. Also, we intend that proteomic analysis should call for specifying and categorizing those molecules. Altogether, these angiocrine molecules work in conjunction with the remaining titanium soluble in the conditioned medium, impacting osteoblast differentiation and thereafter uptaking around 2 ng/mL for up to 7 days in culture, and this might prepare osteoblasts responding to angiocrine molecules concomitantly. In fact, our data support that shear-stressed ECs promote an adequate and bioactive microenvironment, and titanium significantly enhances the expression of genes related to osteoblast differentiation, considering the full mechanism, from early stages by stimulating the expression of transcription factors such as Runx2 and Osterix to bone extracellular matrix remodeling favoring the activity of MMPs.

In agreement, our data supported this hypothesis when the higher expression of the Runx2 gene (6-fold changes) in response to angiocrine molecules in association with the responsiveness to titanium-released molecules was shown. Importantly, it is widely discussed that Runx2 starts the osteoblast differentiation in the early stages of this mechanism [54]. On this hand, and now considering the late stages of osteoblast differentiation, we have shown a dependency of shear-stressed ECs as a source of angiocrine signaling in mature osteoblasts by up-modulating the expression of ALP, BSP, and osteocalcin genes [55]. Altogether, this set of data validates the significance of mechanotransduction-related endothelial cell signaling in providing a bioactive and osteogenic microenvironment to support the maturation of osteoblasts, and it is expected to gain value thereafter through permanent cell cycle withdrawal [56,57]. In detail, the expression profile of ALP reached an amount higher than 150-fold changes when osteoblasts were exposed to angiocrine molecules in conjunction with titanium. In the same context, the BSP profile reached approximately 80-fold changes. This physiological osteogenic condition was acquired considering that the synergism between angiocrine molecules and titanium is unquestionable, and might explain the performance of titanium during the osseointegration mechanism.

Moreover, bone growth and modeling requires finely regulated bone ECM remodeling [11,56,58,59]. In order to better address this issue, we firstly investigated whether this bioactive microenvironment might affect the expression of genes related to collagen synthesis. Our data show that shear-stressed EC contributes to Col3A1 gene expression in osteoblasts. Conversely, non-stressed EC modulates the expression of the Col1A1 gene. Importantly, type III collagen has been correlated in regulating osteoblastogenesis and consequently impacts the quality and amount of the trabecular bone [60]. It makes sense if considering the originality of the bone growing in contact with dental implants independently, either maxillary or mandible—the majority of the bone interacting with implant surfaces is trabecular or bone marrow [61]. Additionally, if osseointegration is strongly influenced by bone quality at the implant insertion site, titanium seems to recapitulate the originality of the histology of bone tissue during the wound healing process in the peri-implant microenvironment, and it distinguishes this mechanism as a regeneration process, looking to avoid the implant’s failure. Moreover, although playing a minimal role considering the primary stability, the trabecular bone certainly plays an important role for implant bone healing and compromises its osseointegration.

This regenerative mechanism also requires finely-modulated ECM remodeling, and in this way, we have also investigated the ability of angiocrine molecules in conjunction with titanium in modulating the expression and activities of MMPs. In this regard, our data clearly show that both MMP2 and MMP9 genes were higher in osteoblasts responding to this bioactive microenvironment. Moreover, this profile of expression reflects the activities of both MMPs. In addition, this bone ECM remodeling seems to have been finely modulated by the requirement of tissue matrix metalloproteinases inhibitors 1 (TIMP1) and TIMP2, once these genes have presented a very similar profile of involvement. The physiological cues of ECM remodeling reveal novel perspectives to tentatively change the cell anchorage on the substrate, and more insights can be gathered to propose mechanotransduction-related endothelial cell signaling as a protagonist of stages coordinating the crosstalk between EC and pOb during bone appositional growth at the peri-implant environment.

## 5. Conclusions

Altogether, our data gather for the first-time sufficient evidence to bring value in mechanotransduction-related endothelial cell signaling as a prerequisite and source of angiocrine molecules, driving paracrine osteoblast differentiation and compromising osteogenesis during bone healing from behind the osseointegration mechanism of dental implants. Furthermore, when these findings are taken together, they can explain the success of titanium as a metallic alloy in biomedical applications.

## Figures and Tables

**Table 1:** Genes, primer sequences, and running conditions.

Gene	Primer	5′-3′ Sequence	Work Conditions
Runx2	Forward	GGACGAGGCAAGAGTTTCA	95 °C—15 s; 60 °C—30 s; 72 °C—60 s
	Reverse	TGGTGCAGAGTTCAGGGAG	
Osterix	Forward	CCCTTCCCTCACTCATTTCC	95 °C—15 s; 60 °C—30 s; 72 °C—60 s
	Reverse	CAACCGCCTTGGGCTTAT	
ALP	Forward	GAAGTCCGTGGGCATCGT	95 °C—15 s; 60 °C—30 s; 72 °C—60 s
	Reverse	CAGTGCGGTTCCAGACATAG	
Osteocalcin	Forward	AGACTCCGGCGCTACCTT	95 °C—15 s; 60 °C—30 s; 72 °C—60 s
	Reverse	CTCGTCACAAGCAGGGTTAAG	
BSP	Forward	GTACCGGCCACGCTACTTTCT	95 °C—15 s; 60 °C—30 s; 72 °C—60 s
	Reverse	GTTGACCGCCAGCTCGTTTT	
Col1a1	Forward	ATGACGTGATCTGTGACGAGAC	95 °C—15 s; 60 °C—30 s; 72 °C—60 s
	Reverse	TTCTTGGTCGGTGGGTGAC	
Col3a1	Forward	GACCTGAAATTCTGCCATCC	95 °C—15 s; 60 °C—30 s; 72 °C—60 s
	Reverse	GCATGTTTCCCCAGTTTCC	
Mmp2	Forward	AACTTTGAGAAGGATGGCAAGT	95 °C—15 s; 60 °C—30 s; 72 °C—60 s
	Reverse	TGCCACCCATGGTAAACAA	
Mmp9	Forward	TGTGCCCTGGAACTCACACGAC	95 °C—15 s; 60 °C—30 s; 72 °C—60 s
	Reverse	ACGTCGTCCACCTGGTTCACCT	
Timp1	Forward	ATCCTCTTGTTGCTATCACTG	95 °C—15 s; 60 °C—30 s; 72 °C—60 s
	Reverse	GGTCTCGTTGATTTCTGGG	
Timp2	Forward	GCAACAGGCGTTTTGCAATG	95 °C—15 s; 60 °C—30 s; 72 °C—60 s
	Reverse	CGGAATCCACCTCCTTCTCG	
Gapdh	Forward	AGGCCGGTGCTGAGTATGTC	95 °C—15 s; 60 °C—30 s; 72 °C—60 s
	Reverse	TGCCTGCTTC ACCACCTTCT	

## Data Availability

The data that support the findings of this study are available from the corresponding author upon reasonable request.
